# Supplementary material for: A solution-to-solid conversion chemistry enables ultrafast-charging and long-lived molten salt aluminium batteries
Source: Nat Commun. 2023 Jul 3;14:3909. doi: 10.1038/s41467-023-39258-y (PMC10318033; doi:10.1038/s41467-023-39258-y)
Supplement: Supplementary file 1 — Supplementary Information [file 41467_2023_39258_MOESM1_ESM.pdf]

SUPPLEMENTARY INFORMATION

A solution-to-solid conversion chemistry enables ultrafast-charging and long-lived molten salt aluminium batteries

Jiashen Meng[1], Xuhui Yao[2], Xufeng Hong[1], Lujun Zhu[1], Zhitong Xiao[1], Yongfeng Jia[1], Fang Liu[1], Huimin Song[1], Yunlong Zhao[3], Quanquan Pang[1]*

1. Beijing Key Laboratory of Theory and Technology for Advanced Batteries Materials, School of Materials Science and Engineering, Peking University, Beijing, 100871, China

2. Advanced Technology Institute, Department of Electrical and Electronic Engineering, University of Surrey, Guildford, GU2 7XH, UK

3. Dyson School of Design Engineering, Imperial College London, London SW7 2BX, UK

*Correspondence: qqpang@pku.edu.cn

Table of Contents

Supplementary Fig. 1. Morphology characterizations of the indium foil before and after treatment in the chloroaluminate melt........ **S3**

Supplementary Fig. 2. Experimental evidences of the spontaneous reaction between indium foil and the chloroaluminate melt electrolyte........ **S4**

Supplementary Fig. 3. Characterizations of the Al deposit on Mo foil........ **S5**

Supplementary Fig. 4. Characterizations of the carbon cloth (CC) and activated carbon cloth (ACC) current collectors........ **S6**

Supplementary Fig. 5. Electrochemical characterizations of the cells using different current collectors and electrolytes........ **S7**

Supplementary Fig. 6. XPS measurement on the ACC cathode of Al|ACC/In cell before cycling and after full charge........ **S8**

Supplementary Fig. 7. Structure characterizations of the cells after full charge........ **S9**

Supplementary Fig. 8. Further analysis of the *in-situ* EIS measurement on the Al|ACC/In cell........ **S10**

Supplementary Fig. 9. The self-discharge test of the Al|ACC/InCl cell operating at 150 °C. **S11**

Supplementary Fig. 10. Further demonstration of the electrochemical performances of the Al|ACC cell and Al|ACC/InCl cell........ **S12**

Supplementary Fig. 11. The rate performance of the Al|ACC/InCl cells with high InCl mass loadings........ **S13**

Supplementary Fig. 12. The extension of the solution-to-solid conversion chemistry for other redox couples........ **S15**

Supplementary Fig. 13. Evaluation of the Al-InCl cells using practical evaluation measures of high areal capacity cycling and freeze-thaw stability tests........ **S16**

Supplementary Table 1. The aluminium and indium concentrations of the electrolytes with different added components after heat treatment at different temperatures........ **S17**

Supplementary references........ **S18**

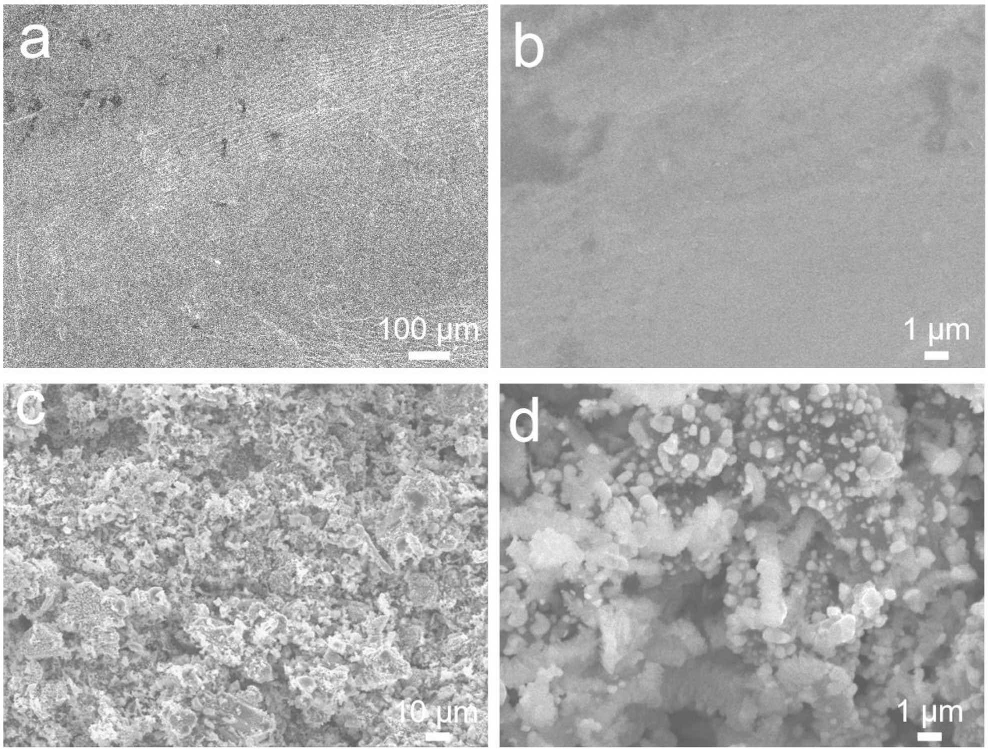

Supplementary Fig. 1. Morphology characterizations of the indium foil before and after heating treatment in the chloroaluminate melt. (a, b) The SEM images of the indium foil before treatment. (c, d) SEM images of the indium foil after treatment at 190 °C in the molten salt. Before reaction, the indium foil has smooth surface; after heat treatment, the indium foil shows a rough surface with abundant aggregated particles, indicating the occurrence of a substantial reaction between the metallic indium and chloroaluminate melt.[1]

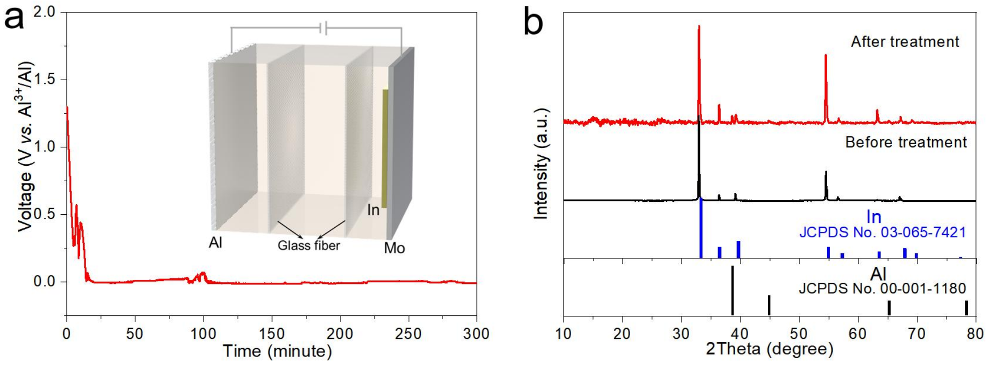

Supplementary Fig. 2. Experimental evidences of the spontaneous reaction between indium foil and the chloroaluminate melt electrolyte. (a) The open circuit voltage-time trace of an Al-In@Mo cell that has an In foil physically contact the Mo cathode current collector (different than the cell configuration in Fig. 2e, main text). Inset of (a) is the schematic illustration of the configuration of this Al-In@Mo cell. When the indium foil was put on the Mo current collector, the assembled Al-In@Mo cell showed a fast voltage decay to near 0 V in open circuit state because of the formation of Al on the In@Mo side. (b) XRD patterns of the indium foil before and after being placed in the assembled Al-In@Mo at 150 °C in the Al-In/Mo cell. The XRD patterns further confirm the formation of Al on indium foil after treatment. These results confirm the spontaneous reaction between indium foil and chloroaluminate melt.

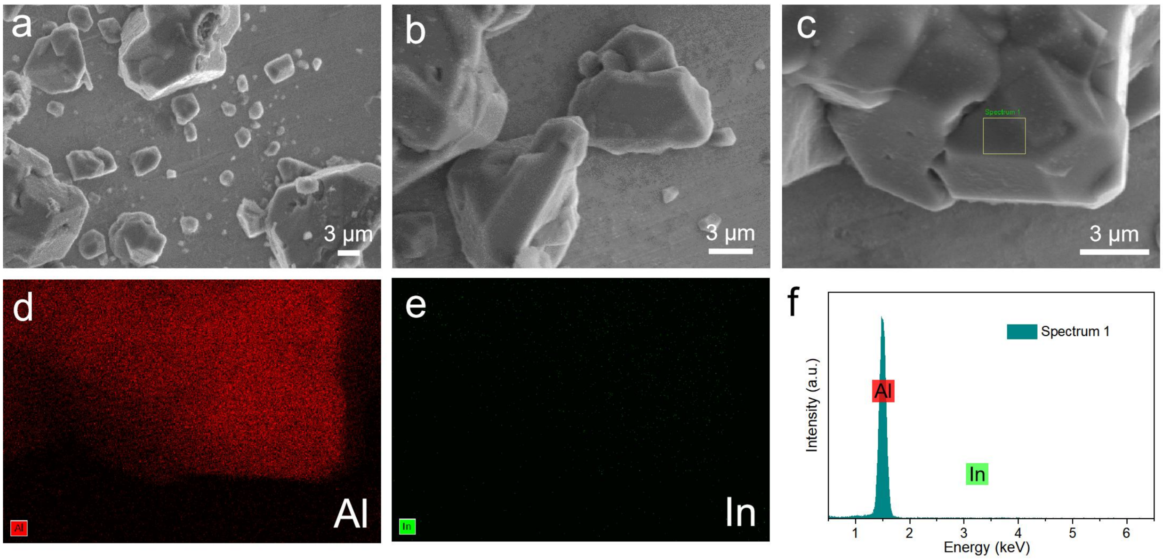

Supplementary Fig. 3. Characterizations of the Al deposit on Mo foil. The SEM images (a-c) and the corresponding EDX mapping images (d, e), and EDX spectrum (f) of plated Al (on Mo substrates) performed in the NaCl-KCl-$AlCl_3$ electrolyte at 150 °C with the current of 1 mA cm^{-2} for an areal capacity of 5 mAh cm^{-2}. After deposition (1 mA cm^{-2}, 5 mAh cm^{-2}), the Al deposits on Mo foil show compact crystals with well-defined facets. The EDX spectra and mapping images show that there is none indium signal on the Al deposits to the resolution and detection limit of our EDX detector. This clearly shows that the Al deposit is exclusively Al metal *via* a three electron reaction. In principle, the active indium metal can spontaneously reduce the chloroaluminate species to generate soluble monovalent In^+ and Al because of a more negative In(I)/In(0) couple than the Al(III)/Al(0) couple. Therefore, the formation of pure Al deposit is thermodynamically driven during reaction.

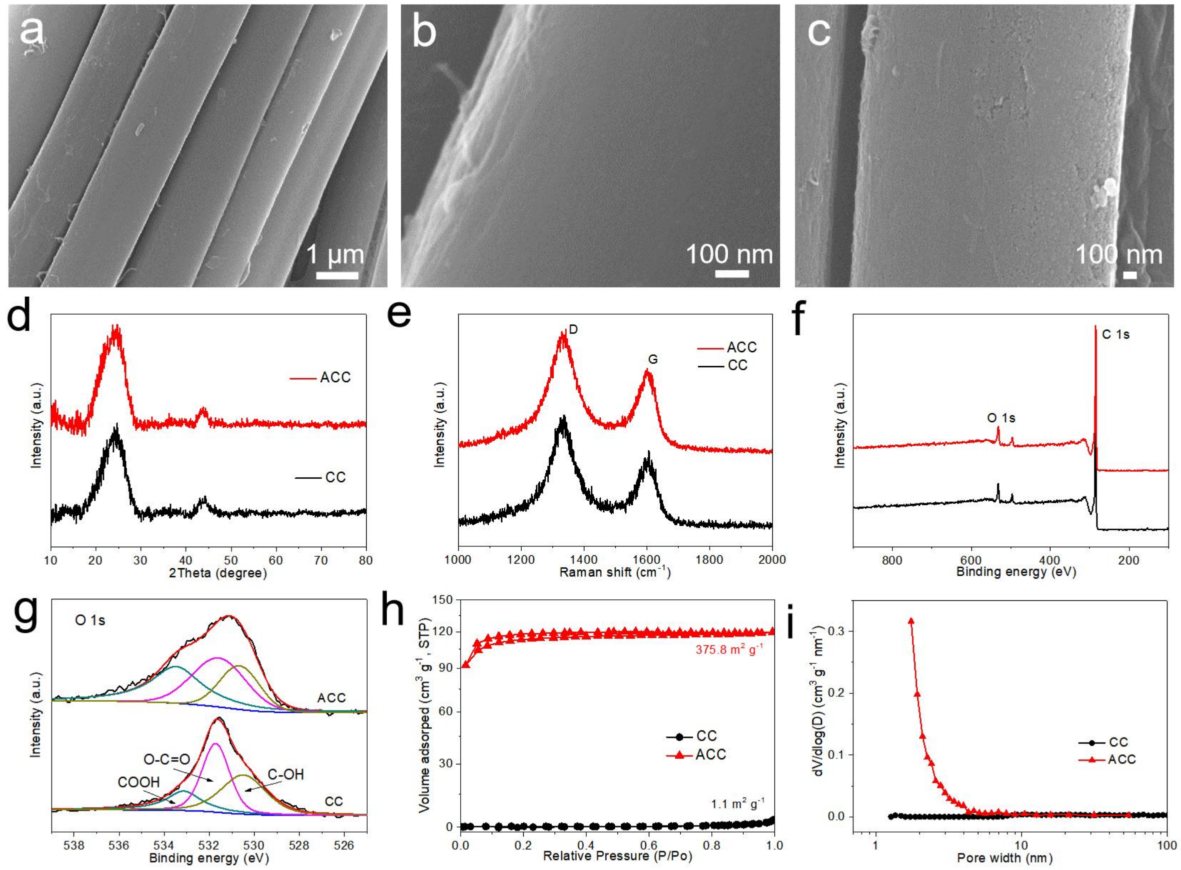

Supplementary Fig. 4. Characterizations of the carbon cloth (CC) and activated carbon cloth (ACC) current collectors. (a, b) SEM images of the CC current collector. (c) SEM image of the ACC current collector. (d, e) XRD patterns and Raman spectra of the ACC and CC current collectors. (f, g) XPS full spectra and high-resolution O 1s XPS spectra of the ACC and CC current collectors. (h, i) The Brunauer-Emmett-Teller (BET) isotherms and Barrett-Joyner-Halenda (BJH) pore size distribution curves of the ACC and CC current collectors. After a facile calcination in air, the resulting ACC current collector exhibits rough surface and abundant pinholes due to the partial oxidization of surficial carbon.[2, 3] The high surface area is beneficial for the surface-precipitation reaction of indium conversion chemistry and thus allows us to achieve high capacity.

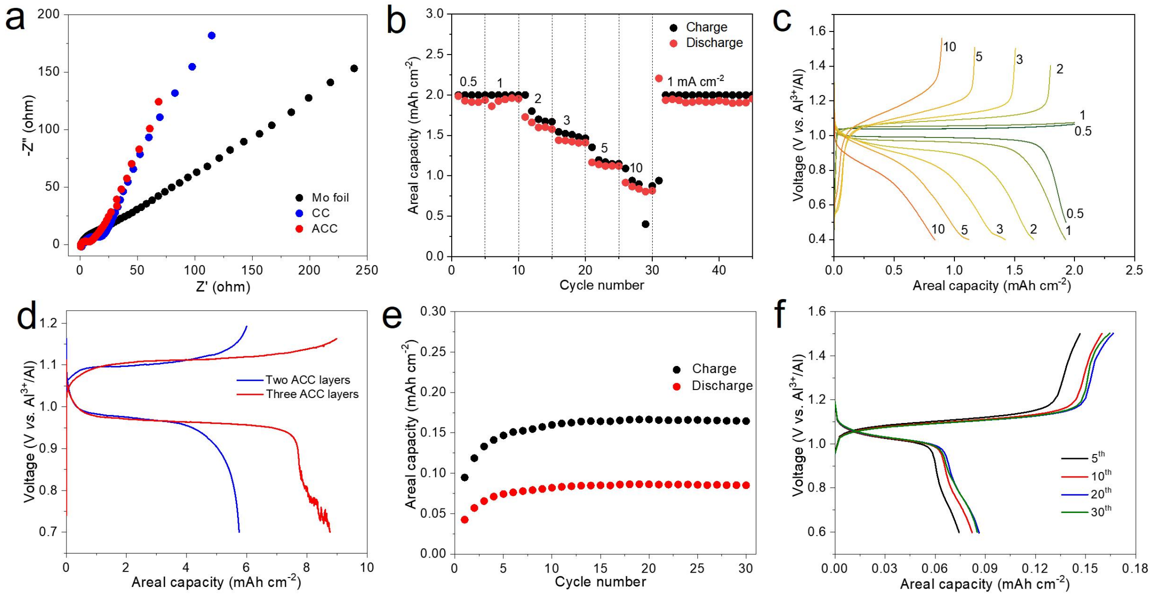

Supplementary Fig. 5. Electrochemical characterizations of the Al-In cells using different current collectors and electrolytes. (a) EIS curves of the Al|Mo/In, Al|CC/In and Al|ACC/In cells using the molten salt electrolytes at pristine state. (b) Rate performance of the Al|ACC/In battery at different rates ranging from 0.5 to 10 mA cm^{-2}, and (c) the corresponding voltage profiles. The cell use same rates for the discharge and charge at all cycle numbers. (d) The voltage profiles of the molten salt Al|ACC/In cells using two and three ACC layers at a charge current of 2 mA cm^{-2} and a discharge current of 1 mA cm^{-2}. Note that the curve fluctuation at a high discharge capacity may be due to local structure conditioning upon discharge. (e) Cycling performance of the Al|ACC/In cell using ionic liquid electrolyte at 1 mA cm^{-2} and 150 °C, and (f) the corresponding voltage profiles at different cycles.

Discussion: The Al|ACC/In cell shows a lower charge-transfer resistance (R_{ct}) than Al|Mo/In and Al|CC/In cells, which can be attributed to the high surface area value of ACC, providing more active reaction sites for fast charging of the cell.[4] The Al|ACC/In cells based on two and three ACC layers showed higher areal capacities of 5.75 and 8.77 mAh cm^{-2} with high CE of above 95%. However, the cell using the ionic liquid electrolyte exhibits a very low discharge capacity and a low CE. This large difference is attributed to unique physicochemical property of the indium foil and indium compound in our inorganic chloroaluminate electrolyte. We thus conclude that use of the high-surface-area current collector and inorganic chloroaluminate electrolytes is necessary to enable the solution-to-solid conversion chemistry with high energy and ultrafast-charging property in RABs.

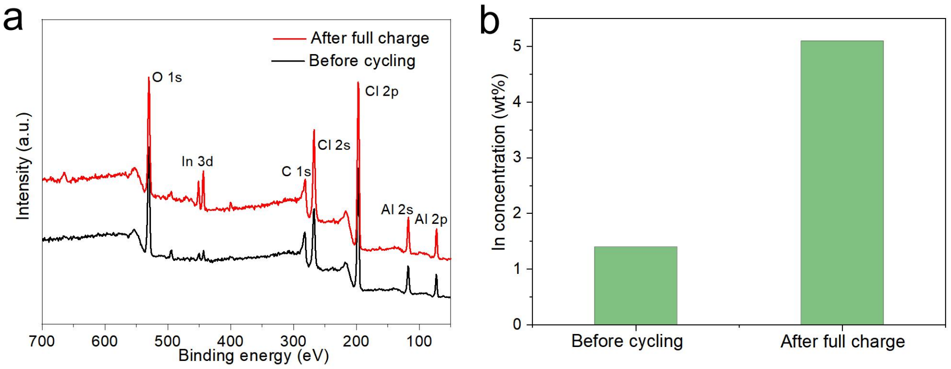

Supplementary Fig. 6. XPS measurement on the ACC cathode of Al|ACC/In cell before cycling and after full charge. (a) XPS full spectra of the cathodes of the Al|ACC/In cell before cycling and after full charge. (b) The corresponding mass fraction of indium in the cathode before cycling and after full charge. As shown in the XPS full spectra, the indium signal greatly increases after full charge. Based on the semi-quantitative result from the surface XPS measurement, the mass fraction of indium on the cathode side rapidly increases from 1.4 wt% to 5.1 wt%, indicating the accumulation of a solid phase $InCl_3$ during charging.

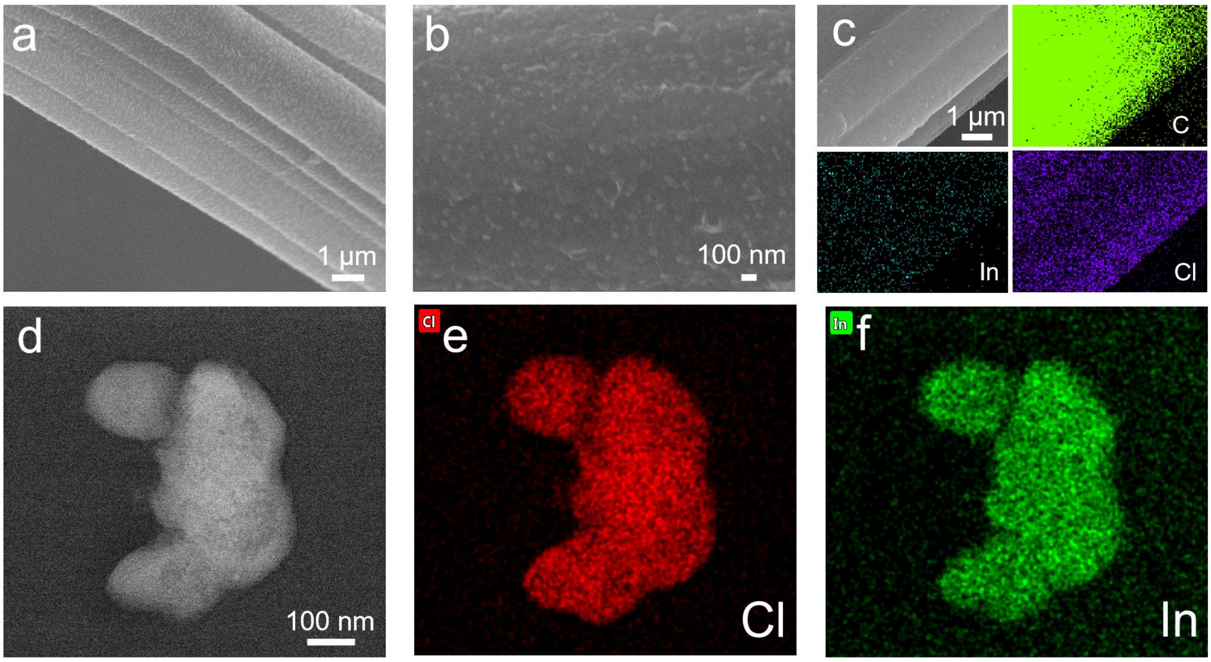

Supplementary Fig. 7. Structure characterizations of the cells after full charge. (a, b) SEM images of the ACC cathode in the Al|ACC/In cell after full charge. The ACC cathode after full charge shows preserved fiber morphology and rough surface covered by abundant solid nanoparticles. (c) A SEM image and the corresponding element mappings of the ACC cathode after full charge, show uniform distribution of the In and Cl elements, confirming a surface-deposition reaction of indium conversion chemistry during charge. (d-f) HAADF-STEM image and the corresponding element mappings of the selected nanoparticles on the cathode, which along with the high-resolution TEM in Figure 4d, e, confirms formation of solid $InCl_3$ on charge.

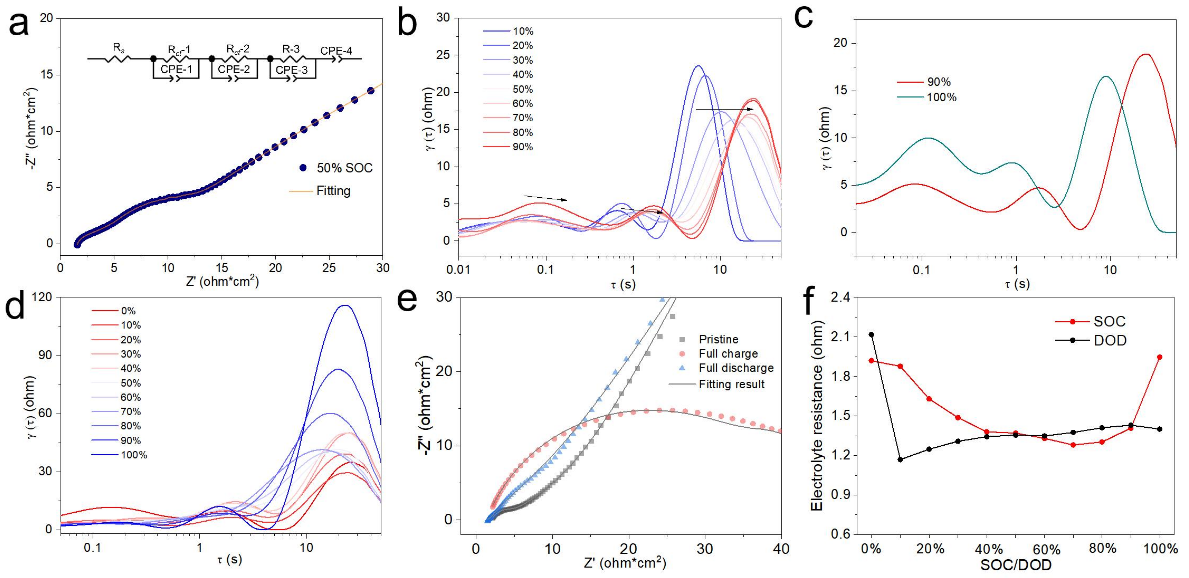

Supplementary Fig. 8. Further analysis of the *in-situ* EIS measurement on the Al|ACC/In cell. (a) The EIS spectrum and corresponding fitting plot of the Al|ACC/In cell at 50% SOC, showing an example of the fitting process and the used equivalent circuit model (Inset). (b, c) The corresponding DRT transformation of the EIS spectra measured in the SOC range (b) from 10% to 90% and (c) from 90% to 100%. (d) The corresponding DRT transformation of EIS spectra measured in the DOD range from 0% to 100%. (e) The magnified EIS spectra measured at the pristine state, after full charge and after full discharge. (f) Plots of the electrolyte resistances R_s in the Al|ACC/In cell during the whole charge and discharge process. DRT, distribution of relaxation time distribution; CPE, constant phase element.

Discussion: As fitted from the Nyquist plot at 50% SOC, three components are required to fit the spectrum, which combined with the DRT analysis we attribute to the R_{ct} of Al anode, the cathode, and a new interphase from the deposited product. *In-situ* EIS spectra and corresponding DRT analysis demonstrate a highly reversible solution-to-solid conversion process in the Al|ACC/In cell, which is consistent with the observations from the *ex-situ* XRD, TEM and XPS characterizations. In addition, the solution resistances vary in a narrow range during the whole charge and discharge process, which are negligibly affected by the variation of the electrolyte compositions.

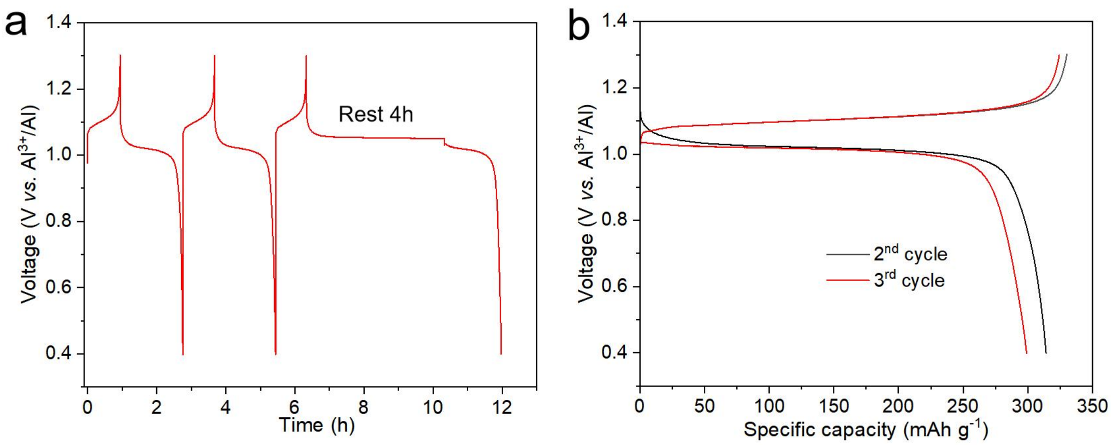

Supplementary Fig. 9. The self-discharge test of the Al|ACC/InCl cell operating at 150 °C. (a) The voltage profile of the Al|ACC/InCl cell during the charge and discharge process with an interval after charging. A charging rate of 1C and discharging rate of 0.5C are performed, consistent with other cycling performance tests of the Al|ACC/InCl cell. (b) The corresponding voltage profiles at the second and third cycles.

Discussion: We have now investigated the self-discharge property of the Al|ACC/InCl cell operated at 150 °C. The cell was cycled in a charging rate of 1C and a discharging rate of 0.5C, showing a high CE of ~95%. The discharge capacity in the second cycle is ~314 mAh g^{-1}. In the third cycle, the cell was charged to 1.3 V at a rate of 1C, kept at 150 °C for 4 h, and then discharged to 0.4 V at a rate of 0.5C. The cell can sustain a discharge capacity of 299 mAh g^{-1}, displaying a high capacity retention of~95.2% compared with the discharge capacity in the second cycle. This indicates that our cell exhibits relatively low self-discharge property even at a high operation temperature, attributed to the low solubility of solid $InCl_3$ in the molten salt electrolyte.

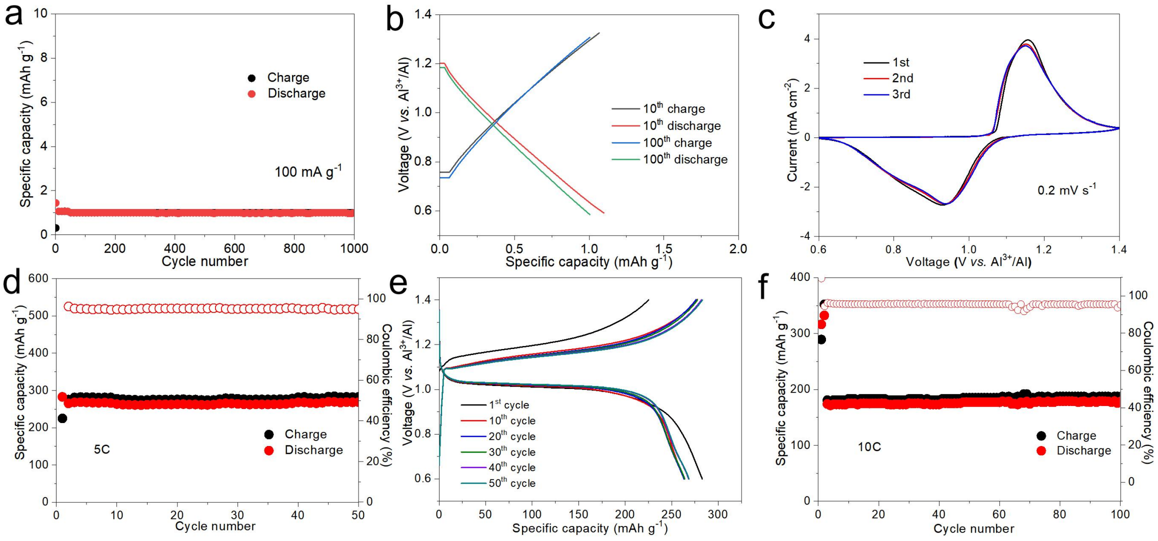

Supplementary Fig. 10. Further demonstration of the electrochemical performances of the Al|ACC cell and Al|ACC/InCl cell. (a) Cycling performance of the Al|ACC cell (without InCl) at a current of 100 mA g_{ACC}^{-1} (a mass loading of ~10 mg for ACC). (b) The corresponding voltage profiles at different cycles. Due to the negligible capacity from the ACC current collector, we conclude that almost all capacity shown in Figures 3 and 5, main text, comes from the solution-to-solid conversion reaction of InCl in the Al|ACC/InCl cell. (c) CV curves of the Al|ACC/InCl cell at 150 °C and a scan rate of 0.2 mV s^{-1}. The close-to-overlapping CV curves in the first three cycles indicate a highly reversible conversion reaction process. (d) The cycling performance of the Al|ACC/InCl cell at a charging rate of 5C (discharging rate of 0.5C) and (e) the corresponding voltage profiles at different cycle numbers. (f) Cycling performance of the Al|ACC/InCl cell at a higher charging rate of 10C (discharging rate of 0.5C).

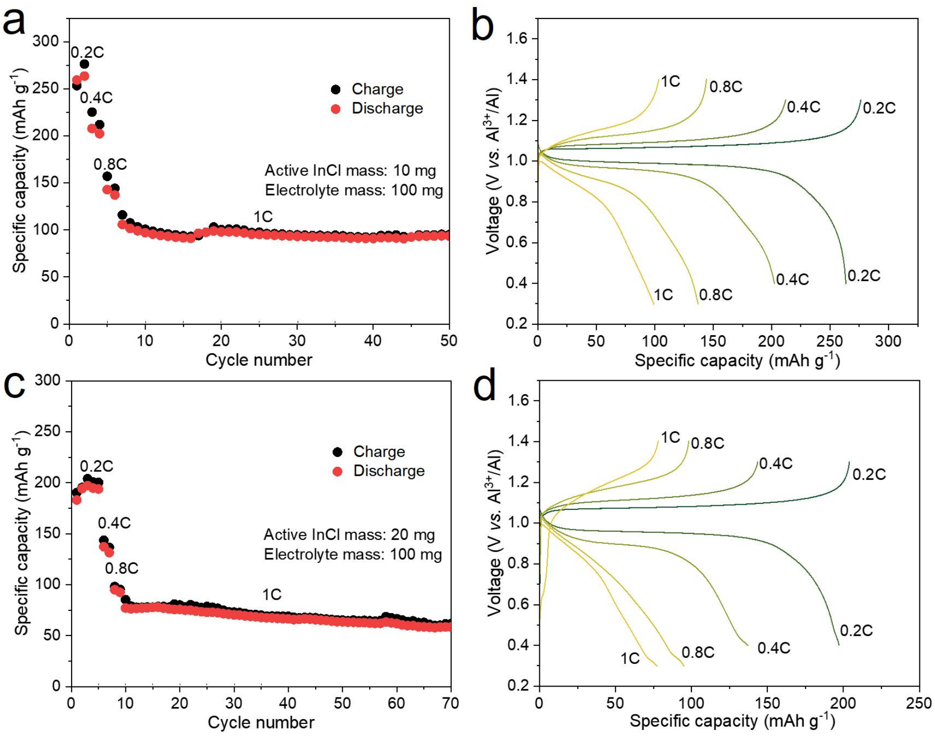

Supplementary Fig. 11. The rate performance of the Al|ACC/InCl cells with high InCl mass loadings. (a) The rate performance of the Al|ACC/InCl cell with active InCl mass of 10 mg and electrolyte mass of 100 mg at 150 °C and varied rates from 0.2C to 1C, and (b) the corresponding voltage profiles. (c) The cell rate performance with active InCl mass of 20 mg and electrolyte mass of 100 mg, and (d) the corresponding voltage profiles.

Discussion: the rate performance of the Al|ACC/InCl cells with high InCl mass loadings and less electrolyte. In the experiment, the mass of molten salt electrolyte added in each cell was fixed to be 100 mg, and the mass of InCl was varied. We first examined the performances using a high InCl loading of 10 mg. At the operation temperature of 150 °C, an InCl mass of 10 mg does not fully dissolve into the electrolyte (based on the measured solubility shown in Fig. 2d). To be exact, about 60% of the active InCl material exists in solid state. As shown in Figure R3a,b, the Al|ACC/InCl cell shows a high discharge capacity of ~261 mAh g^{-1} (compared to 327 mAh g^{-1} capacity at a InCl loading of ~2 mg cm^{-2} shown in Figure 5c), a high Coulombic efficiency of ~96% and small overpotential of ~25 mV at a rate of 0.2C. The high InCl utilization rate obtained shows that even if 60% of the InCl is present as solid, high InCl utilization of 73% can be achieved. When increasing the current density to 1C, the cell exhibits a reversible capacity of ~100 mAh g^{-1} and good cycling stability. Given that such high loading can experience capacity limitation from the mass diffusion problem, it is thus not possible to isolate and determine the limits from the reaction kinetics; nevertheless, the

utilization rate of 73% at 0.2C can be considered to be high. This experiment proves that not all In(I) has to be dissolved to participate in the solution-to-solid reaction, and it is possible to have solid-to-solution and solution-to-solid reactions occurring in tandem or in parallel at high charging rates.

We also further show that even at a higher InCl mass loading of 20 mg, wherein ~80% of active InCl material exists in solid state, the Al|ACC/InCl cell shows a high specific capacity of ~200 mAh g^{-1} at 0.2 C. At a high charging rate of 1C, the cell can sustain a capacity of 75 mAh g^{-1}. We envision that in such case, we are observing a higher percentage of solid-to-solid reaction which is relatively slow and causes higher voltage polarization.

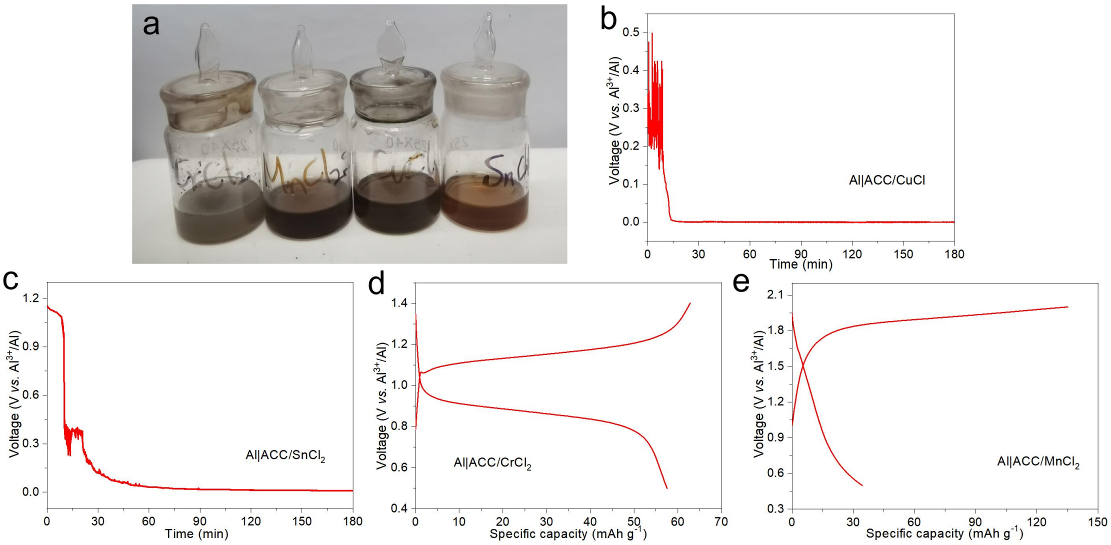

Supplementary Fig. 12. The extension of the solution-to-solid conversion chemistry to other redox couples. (a) The digital photo of the $AlCl_3$-NaCl-KCl chloroaluminate melts with the varied active materials (5 wt% of $CrCl_2$, $MnCl_2$, CuCl and $SnCl_2$). (b, c) The voltage-time traces of (b) the Al|ACC/CuCl cell and (c) the Al|ACC/$SnCl_2$ cell at the open-circuit state. (d, e) The voltage profiles of (d) the Al|ACC/$CrCl_2$ cell and (e) the Al|ACC/$MnCl_2$ cell during the first cycle at a current of 0.1 A g^{-1}. Among them, the Al|ACC/$CrCl_2$ cell shows well-defined voltage plateau with high CE, offering an example on extension of the solution-to-solid conversion chemistry to other metal couples.[5]

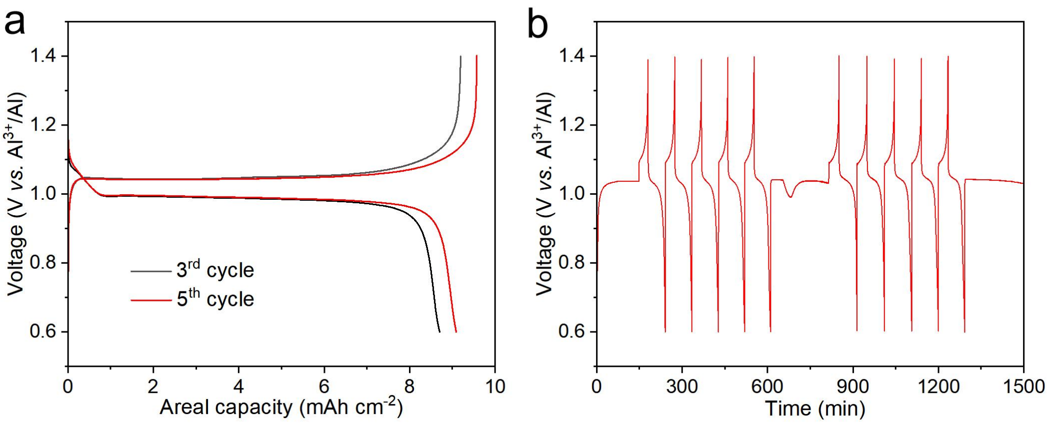

Supplementary Fig. 13. Evaluation of the Al-InCl cells using practical evaluation measures of high areal capacity cycling and freeze-thaw stability tests. (a) The charge-discharge curves of an Al|CNF/InCl cell using a charging rate of 1 mA cm^{-2} and a discharging rate of 0.5 mA cm^{-2}; the battery uses a high surface area CNF film as the current collector. (b) The voltage-time trace of the Al|ACC/InCl cell undergoing a freeze-thaw stability test (a discharging rate of 2C and a charging rate of 4C): the operating temperature of the cell was dropped to room temperature of ~25 °C at the fifth cycle (heating off) and recovered to 150 °C after a period of time for further cycling.

Discussion: From Supplementary Fig. 13a, we can see that due to the high surface area and high conductivity of free-standing CNF, the Al|CNF/InCl cell shows high discharge areal capacity, high CE and very small overpotential.[6] We also propose that, by rational design of the current collector of larger surface area with abundant deposited sites, the capacity of the cell can be further improved. From Supplementary Fig. 13b, it is clear that after freezing and thawing, the Al|ACC/InCl cell still shows stable cycling performance.[7] The excellent thermal stability is attributed to the unique structure-breathing solution-to-solid chemistry that is immune to volume expansion and structure degradation that threats the stability of an otherwise solid-to-solid reaction during a freeze-thaw stability test. The thermal stability lays a foundation for developing high-performance molten salt batteries that may operate intermittently in the grid energy storage field.

Supplementary Table 1. The aluminium and indium concentrations of the electrolytes with different added components after heat treatment at different temperatures.

Added components	Electrolyte components	Treatment temperature (°C)	Aluminium Concentration (wt%)	Indium Concentration (wt%)
Indium foil	$AlCl_3$/KCl/NaCl	110	13.14	1.24
Indium foil	$AlCl_3$/KCl/NaCl	130	13.18	2.15
Indium foil	$AlCl_3$/KCl/NaCl	150	12.8	2.68
Indium foil	$AlCl_3$/KCl/NaCl	170	12.79	3.51
Indium foil	$AlCl_3$/KCl/NaCl	190	12.75	5.05
$InCl_3$	$AlCl_3$/KCl/NaCl	150	10.7	0.02
Indium foil	$AlCl_3$/EMIC	150	12.4	0.17

Supplementary references

1. Anders, U. & Plambeck, J. A. Electrochemistry of copper, silver, gold, gallium, indium, and thallium in fused $AlCl_3$–NaCl–KCl eutectic. *Can. J. Chem.* **47**, 3055-3060 (1969).
2. Gu, Y.-J., Wen, W. & Wu, J.-M. Simple air calcination affords commercial carbon cloth with high areal specific capacitance for symmetrical supercapacitors. *J. Mater. Chem. A* **6**, 21078-21086 (2018).
3. Liu, X. et al. Carbon cloth as an advanced electrode material for supercapacitors: progress and challenges. *J. Mater. Chem. A* **8**, 17938-17950 (2020).
4. Wang, S. et al. Electrochemical impedance spectroscopy. *Nat. Rev. Methods Prim.* **1**, 41 (2021).
5. Lantelme, F., Benslimane, K. & Chemla, M. Electrochemical properties of solutions of $CrCl_2$ and $CrCl_3$ in molten alkali chlorides. *J Electroanal. Chem.* **337**, 325-335 (1992).
6. Wang, K. et al. Super-aligned carbon nanotube films as current collectors for lightweight and flexible lithium ion batteries. *Adv. Funct. Mater.* **23**, 846-853 (2013).
7. Wang, K. et al. Lithium–antimony–lead liquid metal battery for grid-level energy storage. *Nature* **514**, 348-350 (2014).
